# Seroprevalence and trend of *Helicobacter pylori* infection in Gondar University Hospital among dyspeptic patients, Gondar, North West Ethiopia

**DOI:** 10.1186/1756-0500-6-346

**Published:** 2013-08-30

**Authors:** Biniam Mathewos, Beyene Moges, Mulat Dagnew

**Affiliations:** 1 Department of Immunology and Molecular Biology, School of Biomedical and Laboratory Sciences, College of Medicine and Health Sciences, University of Gondar, Gondar, Ethiopia; 2 Department of Medical Microbiology, School of Biomedical and Laboratory Sciences, College of Medicine and Health Sciences, University of Gondar, Gondar, Ethiopia

**Keywords:** *H. pylori*, Dyspeptic patients, Seroprevalence

## Abstract

**Background:**

The growing attention given to *H. pylori* is not surprising since this pathogen colonizes more than at least half of the world’s inhabitants. In Ethiopia particularly in Gondar, there is no current study conducted about seroprevalence and trend of the prevalence of *H. pylori*. Therefore the aim of this study was to determine the seroprevalence and its trend of the *H. pylori* in three consecutive years in North Gondar, North West Ethiopia.

**Findings:**

Retrospective study was conducted using data collected from log book of serology laboratory of Gondar University Hospital. We collected data from January 2009 to December 2011 and 1388 subjects were included whose data were registered completely.

Among all of the study subjects, 912 (65.7%) were found to be seropositive. The prevalence in male was 449/679 (66.1%) and in females it was 463/709 (65.3%). The prevalence of *H.pylori* infection was significantly higher (77.0%) in patients whose age is greater than 60 years and the lowest positive age group was between 0–20 in which only 59.1% were positive (X^2^ =14.15,p=0.0146). The seroprevalence was 86.5% in 2009 and it decreased to 51.8% in 2010. But the seroprevalence increased to 61.3% in 2011.

**Conclusion:**

This study showed high seroprevalence of *H .pylori* among the dyspeptic patients in GUH. The trend of the seroprevalence was varied from year to year in the three consecutive years. In general it showed that the seroprevalence has started increasing.

## Background

*H. pylori* is cause of most chronic bacterial infection in the world. As to different seroepidemiologic studies, 50% of adults in the developed countries and 90% of adults in the developing countries were seropositive for *H. pylori*[1]. This currently discovered organism is a spiral shaped gram negative bacilli that is oxidase, catalase and urease positive and grows slowly in culture [2]. The ecological niche of *H. pylori* is the stomach where the organism establishes long term colonization of the gastric mucosa [3].

The bacteria have been implicated for acid peptic disease and today it is regarded as essential factor and also causative agent of gastritis and peptic ulcer disease [4]. Additionally, the organism is classified as a class 1 carcinogen because of its causal relationship to gastric adenocarcinoma, one of the world’s deadliest cancers [5,6].

The growing attention given to *H. pylori* by academicians and clinicians is not surprising since this pathogen colonizes more than at least half of the world’s inhabitants [7] with an evident geographical variation in its epidemiology. This geographical variation is believed to be largely socio economical, age gender, genetic predisposition and sanitation. In Ethiopia particularly in Gondar, there is no current study conducted about the seroprevalence and its trend in consecutive years of *H*. pylori infection. Therefore the aim of this study was to determine the seroprevalence and its trend in three consecutive years of the *H. pylori* infection in three consecutive years in North Gondar, North West Ethiopia.

## Findings

### Research hypothesis

We hypothesized that the seroprevalence of *H. pylori* is greater than 50% in the study area and the trend of the seroprevalence will show increasing year from year.

## Methods

### Study design and area

Restrospective study was conducted using data from serology log book. We took data from the log book starting from January 2009 to December 2011. We collected data of all patients who were suspected for *H. pylori* infection and who visited the GUH serology laboratory for *H. pylori* test. The hospital is found in Gondar town which is located in the North Gondar Zone of the Amhara region. GUH is one of the oldest health institutions in Ethiopia. The hospital provides different inpatient and outpatient services to more than 5 million people in northwest Ethiopia. The study was conducted the in serology laboratory of the hospital.

### Sample size and sampling techniques

A total of 1388 subjects whose data was completely registered were included in the study.

### Data collection, processing and analysis

Data was collected from log book of the serology laboratory in the hospital. Completeness of the data collected was checked. The frequency distribution of variables was done. The data was entered and analyzed using SPSS version 20.

### Laboratory method

The hospital laboratory generated the data by a serological method of detecting the antibodies for *Helicobacter pylori* from serum or plasma. Anti *H. pylori* antibodies of all isotypes (IgG, IgM, IgA) against *H. pylori* were detected by one step rapid test device (dBest *H. pylori* test strip, Ameritech USA). Appearance of color band on the device on both test line and control line was interpreted as positive but if it is only on the control line as negative result.

### Ethical consideration

We obtained approval from Research and Ethics Committee of School of Biomedical and Laboratory Sciences. Official letter were also obtained from the diagnostic director of the hospital to collect the data. All data obtained has been kept confidential.

## Results

### Socio demographic characteristics of the study subjects

Among the total study subjects, 679 (48.9%) were males and 709 (51.1%) were females. The mean age of the study subjects was 28 year and the range was from 95 to 2 years. A majority of patients 466/1388 (33.6%) were young adults in the age range of 21–30 years compared to the other age groups. The lowest patient number (5.3%) was obtained in patients older than 60 years of age (Table 1). The male to female ratio was 1:1.04.

**Table 1:** **Seroprevalence of *Helicobactor pylori *among dyspeptic patients in Gondar University Hospital, from January 2009 to December 2011**

**Variables**	**N (%)**	**Pos**	**Neg**	**X**^**2**^	**P- value**
		**N (%)**	**N (%)**		
**Age in year**				14.15	0.0146
0–20	252(18.2)	149(59.1)	103(40.9)		
21–30	466(33.6)	307(65.9)	159(34.1)		
31–40	256(18.4)	161(62.8)	95(37.1)		
41–50	227(16.4)	154(67.9)	73(32.1)		
51–60	113(8.1)	84(74.3)	29(25.7)		
>60	74(5.3)	57(77)	17(23)		
**Sex**				0.1	0.746
Male	679(48.9)	449(66.1)	230(33.9)		
Female	709(51.1)	463(65.3)	246(34.7)		

### Seroprevalence of *H. pylori* infection among different age and gender

Among all the study subjects, 912 (65.7%) were found to be seropositive and the remaining 476 (34.3%) were found seronegative for *H. pylori*. The prevalence in male was 449/679 (66.1%) and in females it was 463/709 (65.3%). The prevalence of *H. pylori* infection was significantly higher (77.0%) in patients whose age is greater than 60 years and the lowest seropositivity (59.1% ) was obtained in the age group of 0–20 (x^2^ =14.15, p=0.746) (Table 1).

### Trend of the seroprevalence of *Helicobacter pylori* among three consecutive years

**Figure 1:**
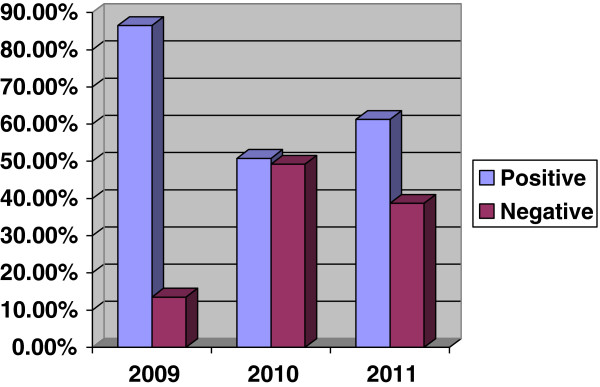
**Trend of seroprevalence of *Helicobacter pylori *among dyspeptic patients from 2009–2011 in Gondar University Hospital, North West Ethiopia.**

The trend of the seroprevalence of *H. pylori* infection among the dyspeptic patients from 2009 to 2011 was that the seroprevalence was 86.5% in the year of 2009 and it decreased to 51.8% in 2010. However, the seroprevalence increased again to 61.3% in the year of 2011 (Figure 1). This trend showed decrement of the seroprevalence temporarily but it started increments after that. This alarm designing appropriate prevention and control strategies is mandatory.

## Discussion

The overall prevalence of *H. pylori* infection in the present study was 65.7% which showed that it was lower than earlier report of prevalence of 85.6% for *H. pylori in* the study area [8]. The low prevalence in the present study may be attributed to improvement in environmental sanitation. When we compare it with other studies it is similar with study conducted in Bahir Dar Felege Hiwot referral hospital (near to Gondar town) with prevalence of (49-70%) [9] and this similarity might be due to the similarity of the two population in socio economic factors, environmental sanitation and their feeding habits.

The present study showed lesser seroprevalence than study conducted in Addis Ababa, capital city of Ethiopia, reported a seroprevalance of 89% [10]. This might be due to elimination of *H. pylori* infection as a result of other antibiotic treatments in occasion of concomitant diseases, such as giardiasis, amoebiasis, and respiratory diseases, etc., as these diseases are reported to be more prevalent in the study area [11].

When we compare the seroprevalence with studies conducted in some other countries like, Iran Hong Kong, United States, Canada (in Ontario) and Kuwait the seroprevalence reported was 43% [12], 42.8% [13], 9.4% [14], 23.1% [15] and 49.7% [16] respectively which all showed lower than the present study. This might be best explained by association between *H. pylori* infection and socioeconomic status since in countries with low socioeconomic status, there is low level of hygiene and environmental sanitation and also in adequate provision of safe water which are known predisposing factors for the infection.

The association between sex and seropositivity in the present study showed that, *H. pylori* infection has no statistically significant association with sex. Similarly in another studies even though there were varying reports of higher prevalence of *H.* pylori infection in either males or females there is no significance association between the seropositivity and sex [8,9].

There was a strong association between age and the disease in this study. Among the six age groups, the lowest (58.8%) and the highest (77.0%) seroprevalence rate were found between the age group of 31-40 years and greater than 60 years age respectively. This result showed that higher frequency of infection was found among older patients. The age related increase in the seroprevalence of the disease in this study was quite similar to findings of previously conducted studies in Inkhorramabad (west Iran) [12], in Kuwait [16] and Kenya [17].

When we see a trend of *H. pylori* infection the prevalence was 86.5%, in 2009 and it showed decrement to 51.8% in 2010. But the seroprevalence resume increasing to 61.3% in 2011. Even though it previously showed some improvements still the seroprevalence is increasing which alarms for designing appropriate prevention and control strategies.

One limitation of our study was we had taken data only from three consecutive years which might reflect the trend of the seroprevalence in a limited way. We couldn’t get data of the year of 2008 and beyond that and this makes us to see the trend of only the three consecutive years.

## Conclusion

This study showed a seroprevalence of 65.7% among the dyspeptic patients in GUH which could be considered a high prevalence. The trend of the seroprevalence was varied from year to year in the three consecutive years. However it is showed that the seroprevalence has started increasing. Further studies in the community which are based on different diagnostic methods such as rapid urase, culture and histological test should be conducted so that the actual situation of *H. pylori* in general population can be known. In addition to this, further epidemiological investigation should be performed in order to determine the source, mode of transmition and the risk factors that might contribute for transmission of the pathogen.

## Abbreviations

GUH: Gondar University Hospital.

## Competing interests

The authors declare that they have no competing interests.

## Authors’ contributions

BM: initiation of the study, design, implementation, analysis and write-up. BM: design, implementation, analysis and write-up. MD: implementation, analysis and write-up. All authors read and approved the final manuscript.

## References

[B1] Mégraud F Epidemiology of *Helicobacter pylori* infection Gastroenterol Clin North Am 1993 22 73 88 8449572

[B2] Goodwin CS Armstrong J Chilvers T Peters M Collins MD Sly L MCConnell W Harper WES Transfer of *Campylobacter pylori* and *Campylobacter mustelae* to *Helicobacter* gen. nov. as *Helicobacter pylori* comb. nov. and *Helicobacter Mustelae* comb. Nov., respectively Int J Syst Bacteriol 1989 39 397 405 10.1099/00207713-39-4-397

[B3] Blaser MJ Ecology of *Helicobacter pylori* in the human stomach J Clin Invest 1997 100 759 762 10.1172/JCI119588 9259572 PMC508245

[B4] Hussain S *H. pylori*. “A multifacet evil” Pak J Gastroenterol 1989 9 2 Editorial

[B5] International Agency for Research on Cancer Infection with Helicobactor pylori IARC Monogr Eval Carcinog Risks Hum 1994 61 177 240 7715070 PMC7681529

[B6] Peter S Beglinger C Helicobacter pylori and gastric cancer: the causal relationship Digestion 2007 75 25 35 17429205 10.1159/000101564

[B7] The EUROGAST Study Group Epidemiology of, and risk factors for, Helicobacter pylori infection among 3194 asymptomatic subjects in 17 populations Gut 1993 34 1672 1676 8282253 10.1136/gut.34.12.1672 PMC1374460

[B8] Feleke M Afework K Getahun M Seroprevalence of *H. pylori* in dyspeptic patients and its relationship with HIV infection, ABO blood groupings and life style in GUH World J Gastroenol 2006 12 1957 1961 10.3748/wjg.v12.i12.1957 PMC4087526 16610007

[B9] Tadege T Mengistu Y Desta K Asrat D Seroprevalence of *Helicobacter pylori* infection in and its relationship with ABO Blood groups Ethiop J Health Dev 2005 19 55 59

[B10] Desta K Asrat D Derbie F Seroprevalence of *helicobactor pylori* infection among health blood donors in Addis Ababa Ethiop Can J Gastroenterol 2007 21 501 506

[B11] Desta Z Abula T Gebre-Yohannes A Worku A Drug prescribing patterns for out patients in three hospitals in northwest Ethiopia Ethiop J Health Dev 2002 16 183 189

[B12] Sheikhian A Ataherian S Delfan M Ebrahimzadeh F Pournia Y Prevalence and risk factor analysis of H. pylori health center referrals in Khorramabad (West Iran) Asian J Epidemiol 2011 4 1 8

[B13] Xia B Xia HHX Ma CW Wong KW Fung FMY Hui CK Chan CK Chan AOO Lai KC Yuen MF Wong BCY Trends in the prevalence of peptic ulcer disease and *H. pylori* infection in Hong Kong Aliment Pharmacol Ther 2005 22 243 249 10.1111/j.1365-2036.2005.02554.x 16091062

[B14] Jackman RP Schlinchting C Carr W Dubois A Prevalence of Helicobacter *pylori* in United States navy submarine crews Epidemiol Infect 2006 134 460 464 10.1017/S0950268805005169 16194289 PMC2576507

[B15] Naja F Kreiger N Sullivan T Helicobacter pylori infection in Ontario: prevalence and risk factors Can J Gastroenterol 2007 21 501 506 17703249 10.1155/2007/462804 PMC2657974

[B16] Alazmi WM Iqbal S Nabeel A Basil A-N Prevalence of *Helicobacter pylori* infection among new outpatients with dyspepsia in Kuwait BMC Gastroenterol 2010 10 14 10.1186/1471-230X-10-14 20128917 PMC2835643

[B17] Haim S Samson O Passaro DJ Galia A Jacob Y Gerald F Silvio P Yaron N Dyspepsia symptoms and *Helicobacter pylori* infection, Nakuru, Kenya Emerg Infect Dis 2003 9 1103 1107 10.3201/eid0909.020374 14519247 PMC3016771

